# F-circEA1 regulates cell proliferation and apoptosis through ALK downstream signaling pathway in non-small cell lung cancer

**DOI:** 10.1007/s13577-021-00628-7

**Published:** 2021-10-11

**Authors:** Yinping Huo, Tangfeng Lv, Mingxiang Ye, Suhua Zhu, Jiaxin Liu, Hongbing Liu, Yong Song

**Affiliations:** 1 Department of Respiratory Medicine, Jinling Hospital, Nanjing Medical University, No 305, East Zhongshan Road, Xuanwu District, Nanjing, 210002 China; 2 Department of Respiratory Medicine, Pukou Branch Hospital of Jiangsu Province Hospital, No 166, Jiangpu Street, Pukou District, Nanjing, 211800 China

**Keywords:** Circular RNA, F-circEA1, EML4-ALK, NSCLC, Apoptosis

## Abstract

Studies have confirmed that circular RNA (circRNA) has a stable closed structure, which plays an important role in the progression of tumors. Cancers with positive fusion genes can produce associated fusion circRNA (F-cirRNA). However, there are no reports concerning a role for F-circRNA of the echinoderm microtubule associated-protein like 4-anaplastic lymphoma kinase variant 1 (EML4-ALK1) in non-small cell lung cancer (NSCLC). Our study confirmed the existence of fusion circEA1 (F-circEA1) in NCI-H3122 cells (carrying the EML4-ALK1 gene), F-circEA1 was expressed both in the cytoplasm and nucleus as determined by fluorescence in situ hybridization (FISH) and Sanger sequencing. CCK8 and transwell assays showed that F-circEA1 was beneficial to cell proliferation, metastasis, and invasion. Overexpression of F-circEA1 can also promote cell proliferation, migration and invasion in A549 and SPCA1 cells (non-small cell lung cancer cell line not carrying the EML4-ALK1 gene). Interference with F-circEA1, induced cell cycle arrest and promoted apoptosis as determined by flow cytometry, and increased drug sensitivity to crizotinib in H3122 cells. F-circEA1 directly affected the expression of parental gene EML4-ALK1. Further research found that F-circEA1 can affect the downstream signaling pathway of ALK. In vivo, the growth rate of xenogeneic tumors was reduced and the protein expression level of EML4-ALK1 was significantly decreased in transplanted tumors measured by immunohistochemistry (IHC) after interference with F-circEA1. In conclusion, F-circEA1 can be considered as a proto-oncogene that regulates cell proliferation and apoptosis by affecting the expression of the parental gene EML4-ALK1 and its ALK downstream signaling pathway in non-small cell lung cancer.

**Supplementary Information:**

The online version contains supplementary material available at 10.1007/s13577-021-00628-7.

## Introduction

Non-small cell lung cancer (NSCLC) remains one of the leading causes of cancer-related death worldwide. Although great progress has been made in treatment regimens including immunotherapy and targeted therapy, the clinical prognosis of NSCLC patients has been little improved due to the recurrence and metastasis of lung cancer and they are still difficult to control [1]. Approximately 85% of lung cancer patients present with NSCLC [2] and patients positive for the EML4-ALK fusion gene represent a subpopulation of these. The EML4-ALK fusion gene is formed by the translocation of EML4 and ALK genes on chromosome 2 [3]. The cleaved terminal ends of EML4 are different and therefore, this makes the fusion site different in ALK, resulting in several EML4-ALK isomers. The most common EML4-ALK fusion variants are 1 and 3, which together account for about 60% of EML4-ALK-positive lung cancer cases [4]. Unfortunately, existing therapies for EML4-ALK-positive NSCLC, such as chemotherapy, radiotherapy, and molecular targeted drugs, have limited efficacy.

In recent years, studies have found that circRNA plays an important role in tumor progression, and cancers with chromosomal translocations can produce associated fusion circRNA, this F-circRNA can affect tumor cell viability, promote cell transformation and have tumor-promoting properties [5]. Recently, it has been found that F-circSR derived from the solute carrier family 34 member A2-reactive oxygen species proto-oncogene 1 (SLC34A2-ROS1) fusion gene, can promote cell migration in NSCLC [6]. F-circEA-2a and F-circEA-4a were found in NCI-H2228 (expressing EML4-ALK variants 3a and 3b [7]) where they are mainly localized in the cytoplasm and promote cell migration and invasion. F-circEA-4a could be a potential liquid biopsy biomarker [8, 9].

Currently, EML4-ALK variant 1 associated F-circRNA and its potential function have not been reported in NSCLC. Our study, using FISH and Sanger sequencing, has found that EML4-ALK variant 1 produced a fused circular RNA termed F-circEA1, which affected tumor proliferation, migration, invasion, apoptosis and cell cycle, and had resistance to the molecularly targeted drug crizotinib in H3122 cells. It has also been found that overexpression of F-circEA1 can also promote cell proliferation, migration and invasion in A549 and SPCA1 cells. Furthermore, it had interesting effects on the expression of the parental gene EML4-ALK1 and its downstream signaling pathway of ALK. Utilizing in vivo studies and the use of a lentivirus to interfere with F-circEA1, we have confirmed that interference with F-circEA1 can inhibit tumor growth and inhibit the expression of the parental oncogene EML4-ALK1. Therefore, our experimental results may provide a new biomarker, treatment method, and prognostic monitoring index for use in the clinic.

## Materials and methods

### Cell lines and cell culture

Three NSCLC cell lines, A549 and SPCA1(no EML4-ALK1 gene), H3122 (EML4-ALK1 positive cells), as well as HBE (human bronchial epithelial cells) were purchased from the Cell Bank of the Type Culture Collection Committee of the Chinese Academy of Sciences and all cells have STR cell identification. All cell lines were tested for mycoplasma contamination before the experiment. The cells were cultured in RPMI 1640 medium (Gibco-BRL, USA)) containing 10% fetal bovine serum (FBS), (Gibco-BRL, USA) and 1% penicillin/streptomycin (Gibco-BRL, USA). Cells were cultured in a humidified incubator containing 5% CO_2_, and at 37 °C.

### Plasmid construction and cell transfection

The pGPU6-F-CircEA1-GFP/Neo (Genepharma, Shanghai, China) was constructed to interfere with F-circEA1 by targeting the back-splicing junction of F-circEA1, and shNC was used as the negative control. The complete sequence of F-circEA1 was inserted into vector pEX3-GCMV-MCS-Neo (Genepharma, Shanghai, China), and an empty vector with no F-circEA1 sequence was used as the control plasmid. All plasmids were transfected with Lipofectamine 3000 (Life Technologies, USA), and RNA and proteins were collected 48 h and 72 h after transfection. The interference sequence and the full-length sequence of F-circEA1 are presented in Supplementary information 1, Data s1.

### RNA or DNA extraction, RNA sequencing and quantification

Total RNA was extracted from cells using Trizol reagent (Life Technologies, USA) and genomic DNA was extracted from H3122 cells using a genomic DNA mini preparation kit with spin columns (Beyotime, Shanghai, China). To digest the linear RNA, 5 µg of total RNA was incubated at 37 °C for 25 min with 20 units of RNase R (Geneseed, Guangzhou, China) and then reverse-transcribed into cDNA with MMLV Reverse Transcriptase (Takara, Dalian, China) according to the manufacturer’s instructions. The cDNA was then amplified using Phanta^®^ Max Super-Fidelity DNA Polymerase (Vazyme, Nanjing, China). The products were electrophoresed on an agarose gel and then sequenced by Invitrogen (Shanghai, China). The relative expression levels of the genes were quantified by QRT-PCR using TB Green^®^ Premix Ex Taq™ (Takara, Dalian, China) and were compared using the 2^−ΔΔCt^ method. The primer sequences involved in the above assays are listed in Supplementary information 1, Data s1.

### RNA nuclear and cytoplasmic isolation and detection

RNAs from the cytoplasm and nuclei were extracted separately with a Cytoplasmic Nuclear RNA Purification kit (Norgen Biotek, Thorold, Canada) according to the manufacturer’s instructions, then reverse-transcribed into cDNA with MMLV Reverse Transcriptase (Takara, Dalian, China), and QRT-PCR using TB Green^®^ Premix Ex Taq™ (Takara, Dalian, China). The relative levels of expression were compared using the 2^−ΔΔCt^ method.

### Fluorescence in situ hybridization (FISH) of F-circEA1 in H3122 cells

Cy3 and DAPI were used to label the back-splice junction (BSJ) (Cy3-5ʹ CAACT + TCATTTGTTGTCA + TGTGTCT-3ʹ-Cy3) of F-circEA1 and cell nuclei, respectively, to observe the location of F-circEA1 in the H3122 cells. FISH was performed according to instructions from the RNA FISH kit (Genepharma, Shanghai, China) and images were taken with a confocal microscope (Carl Zeiss, Goettingen, Germany).

### Cell proliferation and cell viability assays

For the cell proliferation assay, cells were seeded in 96-well plates at a concentration of 3 × 10^3^ cells per well and cultured in complete medium (RPMI 1640 + 10% FBS + 1% penicillin/streptomycin) at 37 °C. Then 10 µL of CCK8 was added into each well at fixed time points for 2 h and the absorbance was measured at 450 nm using a microplate reader (BioTek, Epoch, USA). For the cell viability assays, 5 × 10^3^ cells per well were seeded and cultured in 96-well plates with complete medium at 37 °C for 24 h, then crizotinib (Sigma, Aldrich, USA) or DMSO (as control) (Sigma, Aldrich, USA) was added at different concentrations. After 48 h, 10 µL of CCK8 was added into each well for 2 h and the absorbance read at 450 nm using a microplate reader (BioTek, Epoch, USA).

### Transwell migration and invasion assays

Transwell chambers (Corning, NY, USA) were used for the cell migration and invasion assays. For the invasion assay, the upper chambers were covered with an even layer of Matrigel (BD Biosciences CA, USA) diluted with RPMI-1640 (1:6), but for the migration assay, this step was omitted. The cells were suspended in serum-free RPMI-1640 medium and inoculated uniformly into the upper chamber at a volume of 700 µL and RPMI-1640 medium containing 10% FBS was added to the lower chamber. After incubation at 37 °C, cotton wool swabs were used to carefully remove the cells from the upper surface membrane. The cells on the lower surface of the chamber were fixed with methanol for 15 min and stained with 1% crystal violet (Beyotime, Shanghai, China) for 15 min. At least five different random fields images were chosen to count the cells under the microscope (Carl Zeiss, Oberkochen, Germany).

### Analysis of the cell cycle and apoptosis by flow cytometry

Cells were collected and fixed with 70% ethanol and placed in a refrigerator at 4 °C for 8 h, then stained with propidium iodide (PI) solution for half an hour and then the different cell cycle stages were analyzed within one hour by flow cytometry (Mloflox xdp, Beckman, San Jose, CA, USA). To determine cell apoptosis, cells were stained with Annexin V-PE and 7-AAD according to the protocol within the Annexin V-PE/7-AAD apoptosis detection Kit (Vazyme, Nanjing, China) and then the ratio of apoptotic to healthy cells was measured after one hour.

### Western blot analysis

Cell lysates were prepared with RIPA (Beyotime, Shanghai, China) buffer to isolate total cellular protein. The proteins were then resolved by SDS-PAGE using a 10% gel, and transferred wet to PVDF membranes (Bio-Rad, Hercules, CA, USA). The membranes were blocked with 5% BSA and the following primary antibodies applied overnight at 4 °C: ALK (#3633, D5F3R, 1:2000), p-ALK (#3341, 1:1000), PI3K (#4257, 1:1000), p-PI3K (#4428, 1:1000), AKT (#4691, 1:1000), p-AKT (#5012, 1:2000), mTOR (#2983, 1:1000), p-MTOR (#5536, 1:1000), JAK3 (#8827, 1:1000), p-JAK3 (#5031, 1:1000), STAT3 (#12640, 1:1000), p-STAT3 (#8204,1:2000), MEK1/2 (#8727, 1:1000), p-MEK1/2 (#9154, 1:1000), ERK1/2 (#4695, 1:1000), p-ERK1/2 (#8201, 1:1000), XIAP (#2045, 1:1000), Bac-xl (#2764, 1:1000), Bid (#2002, 1:1000)), Bax (#5023, 1:1000), Cleaved Caspase-3 (#9664, 1:1000), and GAPDH (#5174, 1:1000) (all from Cell Signaling Technology, Beverly, MA, USA). On the next day, blots were incubated with secondary anti-rabbit (1:3000, Cell Signaling Technology, Beverly, MA, USA) at room temperature for 1 h. Finally, protein bands were detected with Immobilob™ Western chemiluminescent HRP substrate (Millipore, Billerica, MA, USA), using a fully automated chemiluminescence imaging analysis system (Tanon 5200, Shanghai, China).

### Establishment of stable cell lines and Nude mice xenografts

Lentiviral interference vector LV3-H1-F-CircEA1-GFP & Puro, and the control lentivirus were constructed by Genepharma (Shanghai, China). After infecting H3122 cells, the transfectants were isolated using puromycin selection to form stable cell lines. Next, 1 × 10^6^ cells and 30 µL Matrigel (BD Biosciences CA, USA) were diluted with PBS to 100 µL, and then inoculated into the armpit of BALB/c nude mice Subcutaneously (5 weeks old, Nanjing Qinglong Mountain Animal Breeding Farm, China), and maintained in a pathogen-free environment. The body weight and tumor volume of the mice were measured every 3 days after tumor formation. Finally, tumorous tissues were collected after 25 days.

### Immunohistochemistry (IHC)

The transplanted tumor tissue was embedded into paraffin and sections were incubated with anti-ALK (D5F3R 1: 250) primary antibody (Cell Signaling Technology, Beverly, MA, USA) overnight at 4 °C, and incubated with secondary anti-rabbit for one hour at 37 °C, then soaked in HRP-labeled antibody/streptavidin solution for 10 min and stained with *diamino*benzidine (DAB). Target protein levels were evaluated based on their intensities within positive cells. Finally, five fields of view were analyzed for each slide, using a fluorescence microscope (Carl Zeiss, Oberkochen, Germany), at a magnification of 400 × . The total score was obtained by multiplying the dye intensity and the percentage of the number of cells. The dye intensity score: 0 points (negative), 1 point (light yellow), 2 points (brown-yellow), 3 points (dark brown). The cell percentages: 0 points (negative), 1 point (1–25% positive cells), 2 points (26–50% positive cells), 3 points (51–75% positive cells), 4 points (76–100% Positive cells).

### Statistical analyses

Statistical analyses were performed using unpaired *t* test or two-way ANOVA test using GraphPad Prism8.0. Differences with a *P* value < 0.05 were considered statistically significant. The grayscale of WB protein was assessed by Image J and the data are presented as means ± standard deviation (SD).

## Results

### Specific expression of F-cirEA1 in EML4-ALK1-positive cells

**Fig. 1:**
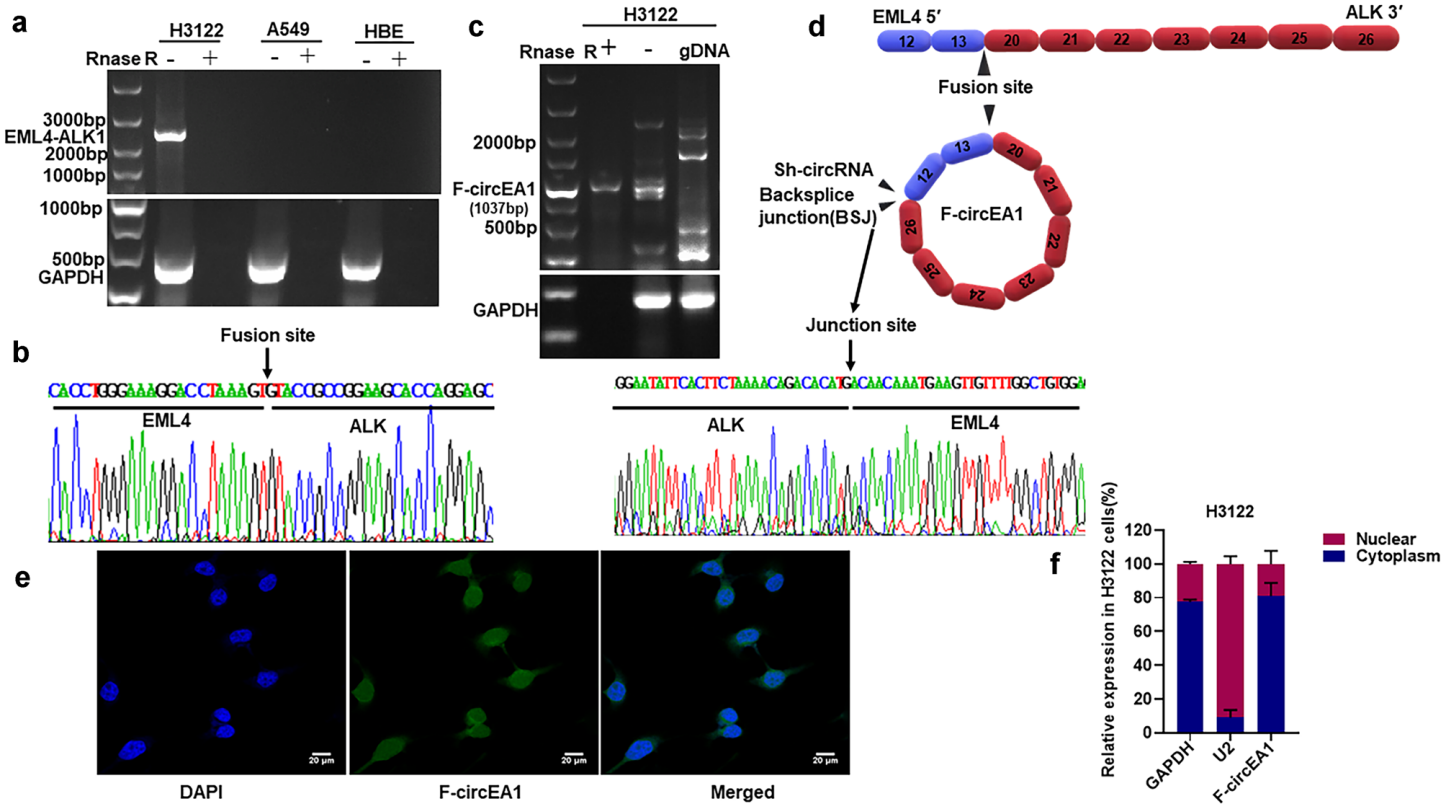
Identification of F-circEA1. **a** EML4-ALK1 and GAPDH were amplified from the cDNA of H3122, A549 and HBE cells, the RNAs of the cells were treated with and without RNase R, respectively, EML4-ALK1 was only expressed in H3122. **b** Sanger sequencing revealed the fusion site of EML4-ALK1. **c** F-circEA1 and GAPDH were detected and amplified from the PCR products of the RNase R-treated, without the RNase R-treated, and the genomic DNA, respectively. F-circEA1 was resistant to RNase R treatment. **d** Sanger sequencing showed the junction site of F-cricEA1. **e** FISH and **f** RNA nuclear and cytoplasmic isolation assay showed that the subcellular distribution of F-circEA1 mainly existed in the cytoplasm. (*n* = 3). (Scale bar = 20 μm)

We first confirmed the expression of EML4-ALK1 in H3122, A549 and HBE (human bronchial epithelial cell) as negative control cells. PCR products confirmed the presence of the EML4-ALK1 gene in H3122 cells only. We also used RNase R to digest linear RNA and found that linear RNA was able to be degraded by RNase R (Fig. 1a). Using the primers F1/R1 from the PCR products and by Sanger sequencing, H3122 cells exhibited the fusion site of EML4-ALK1 (Fig. 1b). Next, the RNA samples were treated with RNase R to remove linear RNA and the divergent primers F2/R2, were used to find the BSJ (the back-splice junction). To prevent the interference of genomic DNA, we also used DNA samples of H3122 cells, only one circular RNA was detected and termed F-circEA1 (Fig. 1c). The primer for GAPDH was F3/R3 as control. The PCR product was identified as the binding site of F-cricEA1, which was formed by reverse splicing of exons 12–13 of EML4 and exons 20–26 of ALK (Fig. 1d). We then went on to design probes for the BSJ, and using fluorescence in situ hybridization (FISH), we found that F-circEA1 was localized both to the nucleus and cytoplasm in H3122 cells (Fig. 1e). Cytoplasmic RNA isolation from H3122 cells confirms that F-circEA1 is mainly expressed in the cytoplasm (Fig. 1f).

### F-circEA1 contributes to the proliferation, migration and invasion of H3122 cells

**Fig. 2:**
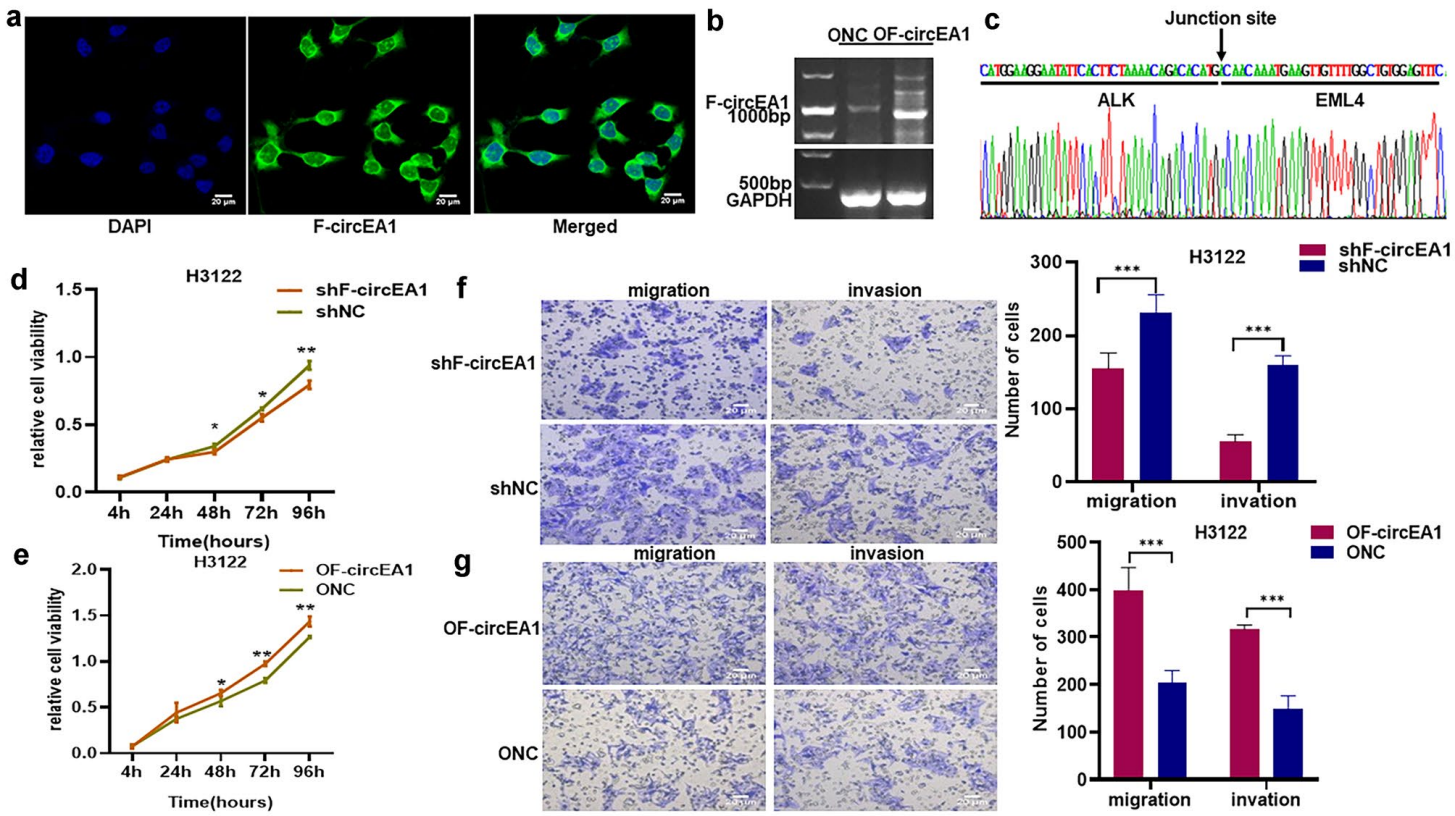
F-circEA1 promotes the proliferation, migration and invasion of H3122 cells. **a** FISH showed F-circEA1 overexpression in H3122. (Scale bar = 20 μm). **b** Agarose gel electrophoresis revealed F-circEA1 overexpression in H3122. **c** Sanger sequencing showed the junction site of overexpressed F-cricEA1. **d** CCK8 assays tested the proliferation of H3122 cells after inhibiting or **e** overexpressing of F-circEA1. (*n* = 3). **f** Images and quantification of the migration and invasion with F-circEA1 interference or **g** overexpression in H3122 cells. (*n* = 5). (Scale bar = 20 μm). (*P < 0.05, **P < 0.01, ***P < 0.005)

To determine a role for F-circEA1 in H3122 cells, we constructed the interference plasmid (pGPU6-F-CircEA1-GFP/Neo) targeting the back-splice junction of F-circEA1 (Fig. s1a), the interference rate of F-circEA1 was 79.09% ± 3.38% after transfection of the knockdown plasmid in H3122 cells (Fig. s1b). We also constructed the F-circEA1 overexpression plasmid containing pEX3-GCMV-MCS-Neo vector (Fig. s1c), the overexpression fold of F-circEA1 was 1883.00 ± 198.23 after transfection of the overexpression plasmid in H3122 cells (Fig. s1d). Overexpression of F-circEA1 was detected in the nucleus and cytoplasm of H3122 cells by FISH and confocal microscopy (Fig. 2a). After overexpression of F-circEA1, agarose gel electrophoresis detection (Fig. 2b) and Sanger sequencing (Fig. 2c) were performed, and confirmed overexpression of F-circEA1 in H3122 cells. Next, CCK8 experiments showed that cell proliferation decreased after interference with F-circEA1 (Fig. 2d), but increased after its overexpression (Fig. 2e). Transwell experiments found that F-circEA1 interference, attenuated the migration and invasion capability of H3122 cells (Fig. 2f) but stimulated them when F-circEA1 was overexpressed (Fig. 2g). These results indicated that F-circEA1 had a proto-oncogenic effect and promoted cancer progression.

### F-circEA1 promotes the proliferation, migration and invasion of A549 and SPCA1 cells

**Fig. 3:**
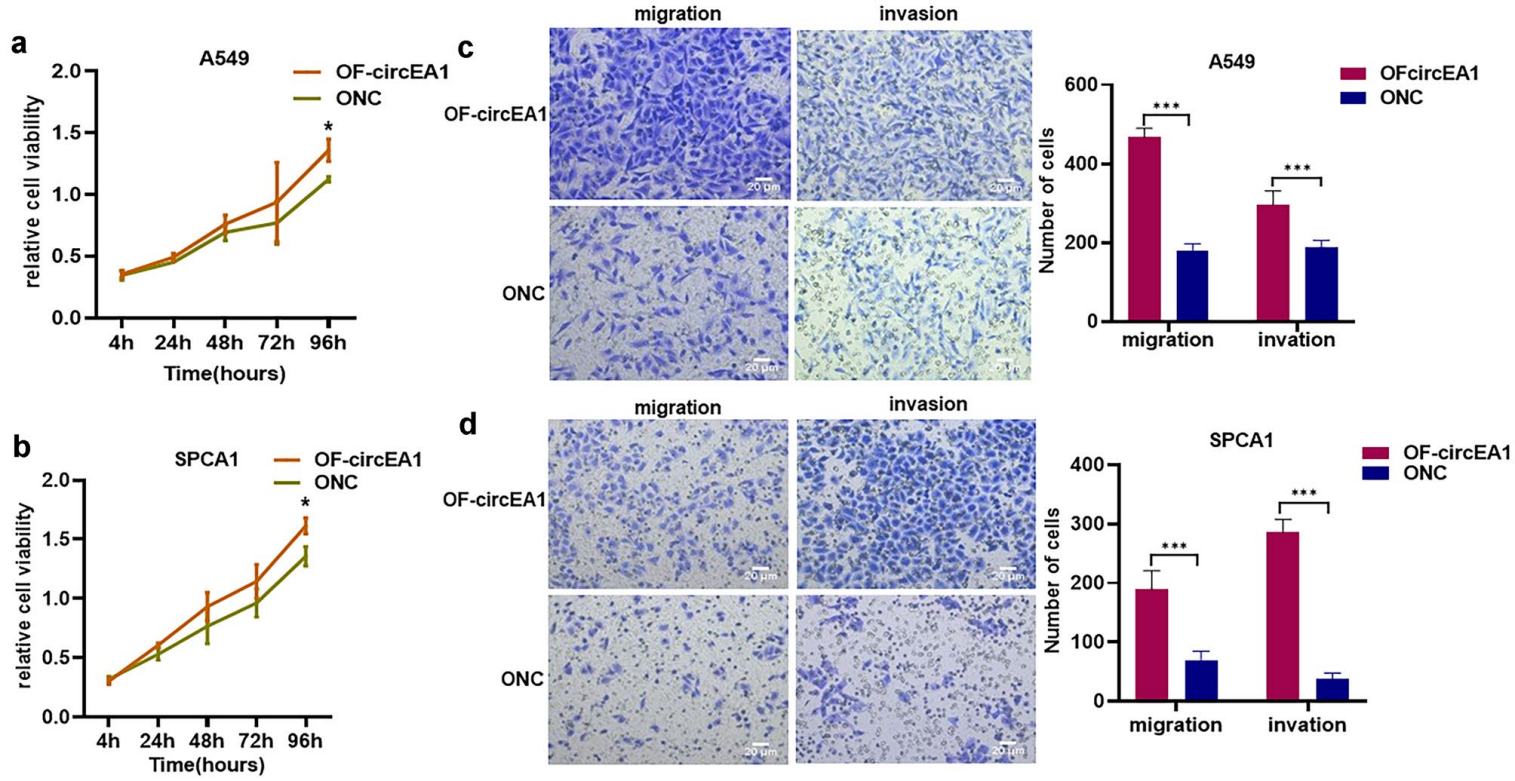
F-circEA1 promotes the proliferation, migration and invasion of A549 and SPCA1 cells. **a** The proliferation of A549 and **b** SPCA1 cells with F-circEA1 overexpression. (*n* = 3). **c** Images and quantification of migration and invasion with F-circEA1 overexpression in A549 and **d** SPCA1 cells. (*n* = 5). (Scale bar = 20 μm). (*P < 0.05, **P < 0.01, ***P < 0.005)

We also transfected F-circEA1 overexpression plasmid in A549 and SPCA1 cells (no EML4-ALK1 gene). CCK8 suggest that F-circEA1 can promote the proliferation of A549 (Fig. 3a) and SPCA1 (Fig. 3b). Transwell experiments found that F-circEA1 overexpression, promoted the migration and invasion capability of A549 (Fig. 3c) and SPCA1 (Fig. 3d) cells. These results showed that F-circEA1 promotes the proliferation, migration and invasion were not dependent on the existence of the EML4-ALK1 fusion gene.

### Interference with F-circEA1 induces cell cycle arrest, promotes apoptosis and drug sensitivity to crizotinib in H3122 cells

**Fig. 4:**
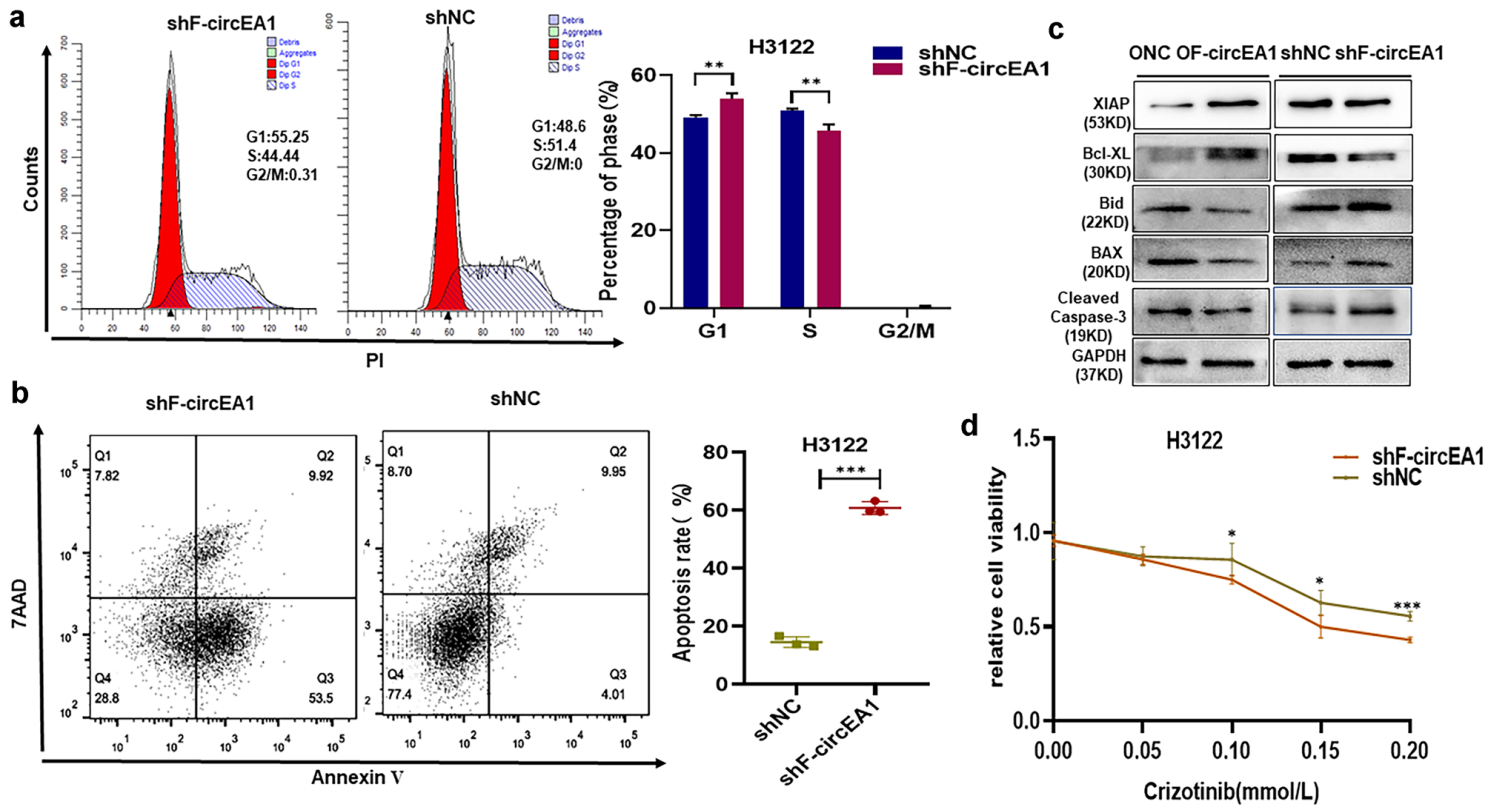
Interference with F-circEA1 induces cell cycle arrest, promotes apoptosis and improves the sensitivity to crizotinib in H3122 cells. **a** Different stages of the cell cycle and **b** the apoptosis rate was quantified in H3122 cells after interference with F-circEA1. (*n* = 3). **c** The effect of overexpression of or interference with F-circEA1 on apoptosis-related proteins. (*n* = 3). **d** Cell viability was assessed with different concentration of crizotinib after cells were subjected to F-circEA1 interference. (*n* = 5). (*P < 0.05, **P < 0.01, ***P < 0.005)

We used flow cytometry to detect the different stages of the cell cycle, and apoptosis in H3122 cells, and found that after interference with F-circEA1, the cell synthesis stage of the cell cycle decreased significantly (Fig. 4a), but apoptosis clearly increased (Fig. 4b). We used western blotting to further detect the expression levels of apoptosis-related proteins and found that inhibition of the apoptosis-related proteins XIAP and Bcl-xl were significantly increased after F-circEA1 overexpression, and pro-apoptosis-related proteins Bax, Bid and Cleaved Caspase-3 were significantly decreased, but interference with F-circEA1 gave the opposite result (Fig. 4c). These findings indicated further that interference with F-circEA1 was not conducive to tumor progression. Crizotinib is a commonly used drug to treat EML4-ALK-positive lung cancer patients. Our CCK8 experimental results showed that H3122 cell viability decreased significantly with increasing concentration of crizotinib and after these cells were subjected to F-circEA1 interference, they showed higher sensitivity to the drug (Fig. 4d).

### F-circEA1 promotes the expression of EML4-ALK1

**Fig. 5:**
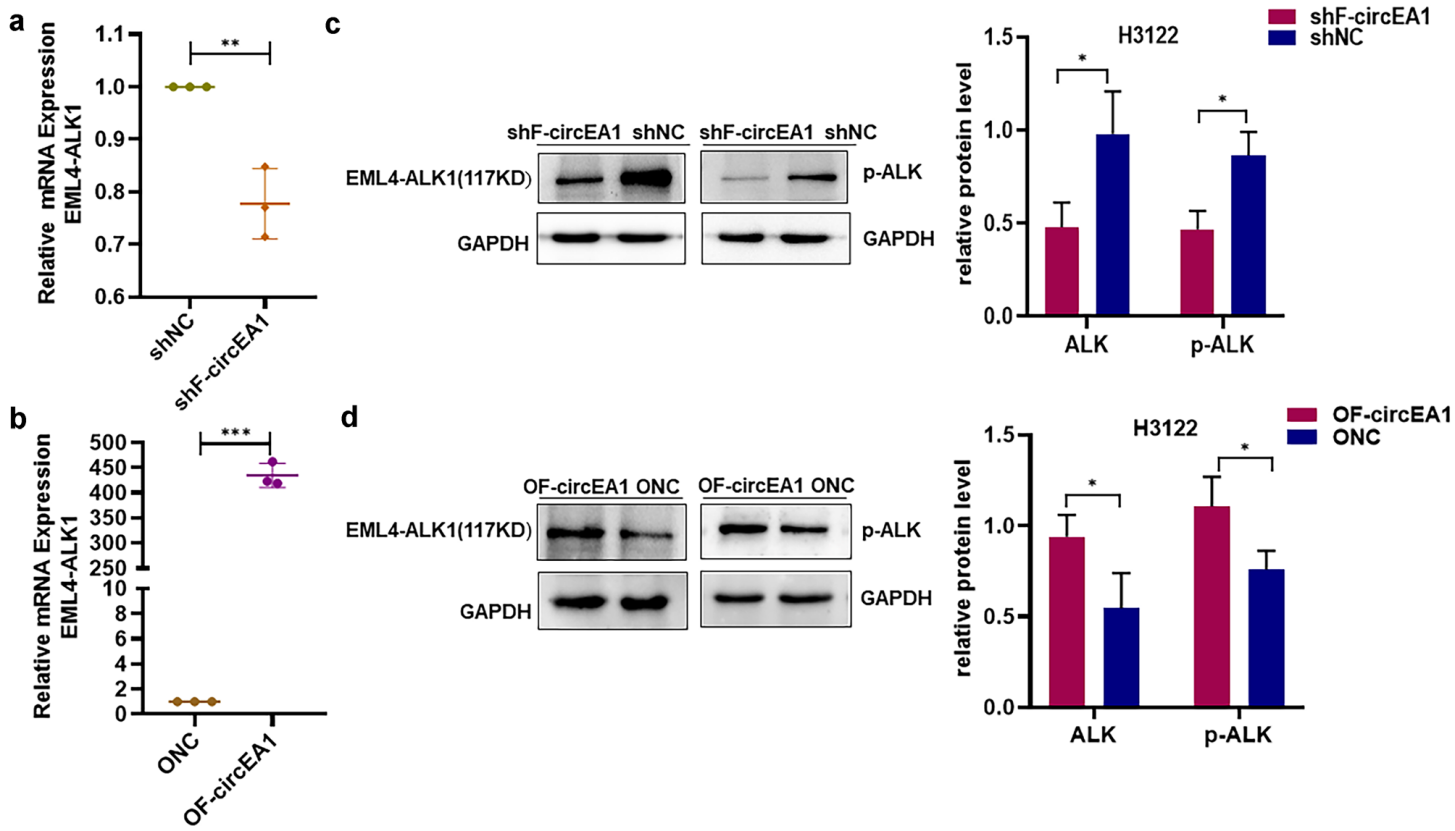
F-circEA1 promotes the expression of EML4-ALK1 in H3122 cells. **a** The mRNA expression of EML4-ALK1 was measured after F-circEA1 interference or **b** overexpression. (*n* = 3). **c** The protein levels of EML4-ALK1 were detected after F-circEA1 interference or **d** overexpression. (*n* = 3). (*P < 0.05, **P < 0.01, ***P < 0.005)

It has been well established that EML4-ALK1 is an oncogene and that F-circEA1 is derived from EML4-ALK1. However, its effects on the expression of EML4-ALK1 are unknown. However, we have detected both mRNA and protein for EML4-ALK1 after interference with and overexpression of F-circEA1 in H3122 cells. The results of QRT-PCR detection using primer F4/R4 showed that the mRNA of EML4-ALK1 decreased after F-circEA1 interference (Fig. 5a), while results were opposite after overexpression of F-circEA1 (Fig. 5b). Next, we used Western blotting to detect ELM4-ALK1 protein in H3122 cells and found that the protein was down-regulated after F-circEA1 interference (Fig. 5c), but up-regulated when F-circEA1 was overexpressed (Fig. 5d). These findings suggest that F-circEA1 may affect the biological function of the H3122 cells by interfering with the expression of EML4-ALK1.

### F-circEA1 activates the downstream signaling pathway of ALK

**Fig. 6:**
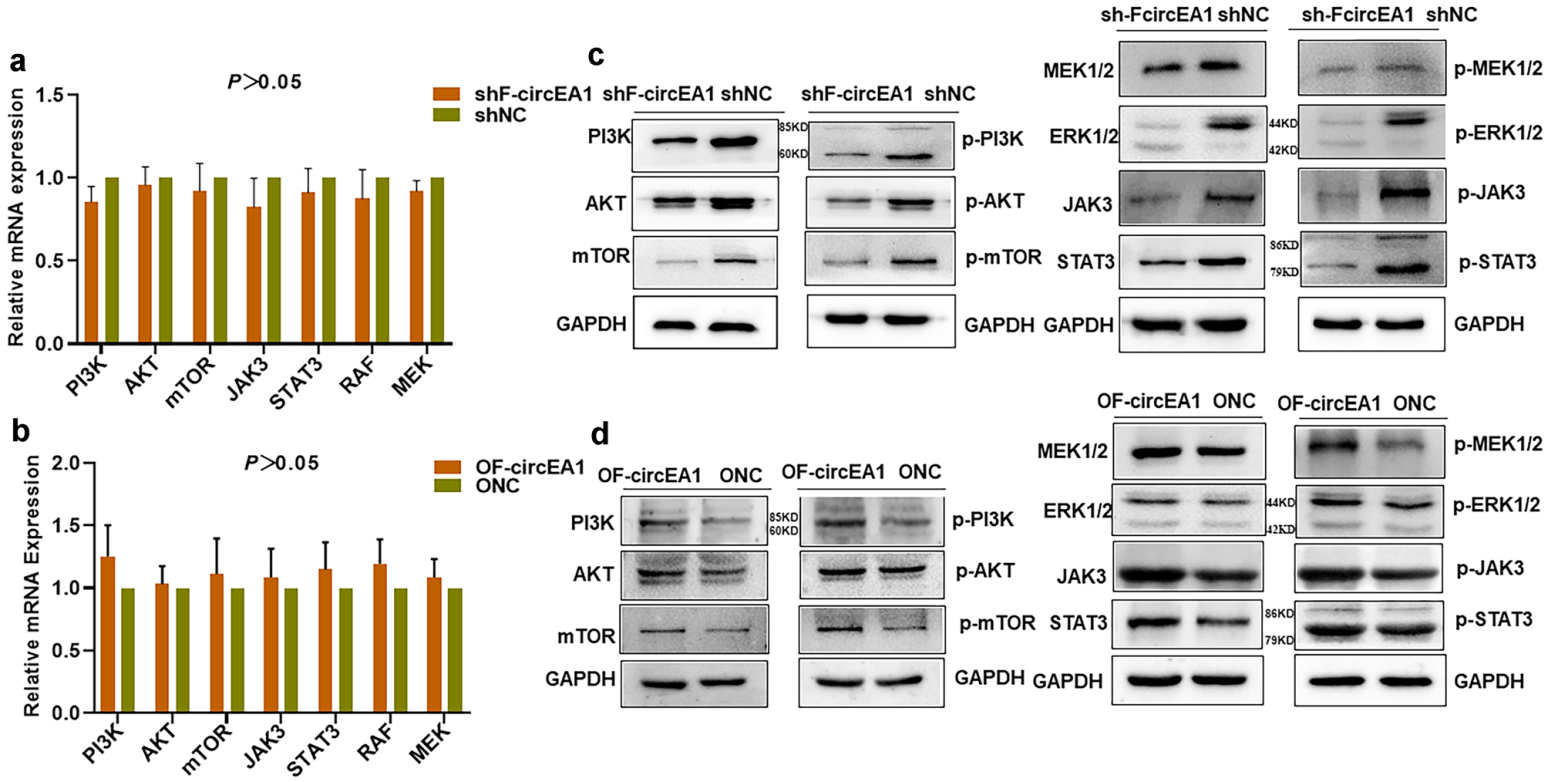
F-circEA1 activates EML4-ALK1 associated downstream signaling pathways in H3122 cells. **a** The mRNA expression of the signaling factors were determined after transfection with F-circEA1 interference or **b** overexpression plasmid. (*n* = 3). **c** The protein expression of the signaling factors were detected after F-circEA1 interference or **d** overexpression. (*n* = 3). (*P < 0.05, **P < 0.01, ***P < 0.005)

It has been reported that the downstream signaling pathways related to EML4-ALK include principally PI3K-AKT-mTOR, JAK3-STAT3, and MEK-ERK1/2 [19]. The mRNA expressions of the above signaling factors were not significantly different after both interference with and overexpression of F-circEA1 by QRT-PCR (Fig. 6a, b). Interestingly, we found that the protein expression of these factors was down-regulated after interference with F-circEA1 but was significantly up-regulated after F-circEA1 overexpression (Fig. 6c, d). It is widely known that these pathways have clear effects on the malignant behavior of tumors, in particular their cell survival, proliferation, and metastasis. Our experimental results showed that F-circEA1 promoted the expression of the parental gene EML4-ALK1, which then activated the downstream signaling pathways related to the parental gene, thereby participating in the malignant biological behavior of lung cancer.

### Interference with F-circEA1 inhibits subcutaneous xenograft growth in nude mice and the expression of EML4-ALK1 protein in their tumors

**Fig. 7:**
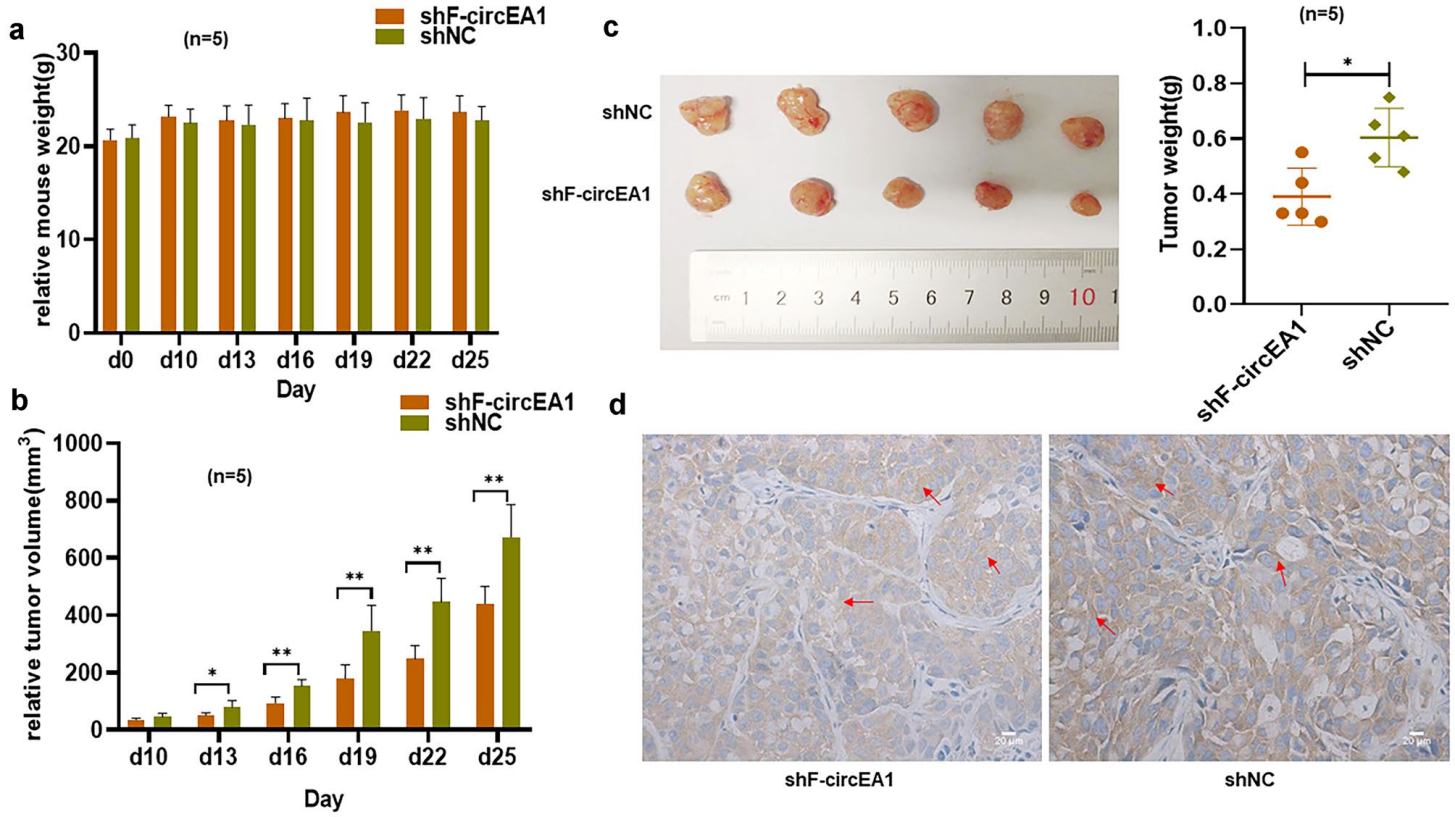
Interference with F-circEA1 inhibited subcutaneous xenograft growth and the protein level of EML4-ALK1 in the tumors. **a** No obvious differences in body weights of the nude mice were seen after interference with F-circEA1 (*n* = 5). **b** Tumor volumes and **c** weights were reduced after interference with F-circEA1 (*n* = 5). **d** EML4-ALK1 protein expressed in the cytoplasm and cell membrane, and EML4-ALK1 protein level decreased in the shF-circEA1 group (*n* = 5). (Scale bar = 20 μm). (*P < 0.05, **P < 0.01, ***P < 0.005)

In vivo, the shF-circEA1 plasmid was inserted into LV3 to form a lentiviral interference plasmid and its transfection diagram in H3122 cells (Fig. s1e, 1sf). These subcutaneous tumor formation experiments in nude mice, showed that interference with F-circEA1 had no obvious detrimental effect on body weight (Fig. 7a) but did significantly inhibit the growth of the subcutaneous xenografts, including volume (Fig. 7b) and weight (Fig. 7c). IHC detected EML4-ALK1 protein as being present in the tumors but this was significantly inhibited after interference with F-circEA1 (Fig. 7d). This is consistent with our cell experiments, which showed that F-circEA1 affected the expression of EML4-ALK1 and participated in tumor proliferation.

## Discussion

CircRNA is different from linear RNA, as both the 5′ cap and 3′ tails are absent, instead it forms a covalently closed loop structure with no 5′–3′ polarity, or polyadenylation tail. Therefore, circRNA is resistant to ribonuclease R treatment [10, 11]. Recently, it has been found that circRNAs play an important role in the pathogenesis of cancer and can affect many characteristics of the disease. In addition, circRNA can be found in body fluids (such as blood and saliva) with non-invasion; therefore, circular RNA has great potential as a cancer biomarker [11–16].

Chromosome translocation is one important carcinogenic driving mechanism in cancer and different types of chromosomal translocations lead to the expression of specific F-circRNAs in related tumor cells [5]. F-circRNA-hC, F-circRNA-hD, F-circRNA-hE, and F-circRNA-hF have all been found in nucleophosmin 1-anaplastic lymphoma kinase (NPM1-ALK)-positive fusion gene tumors [17]. F-circPR can be found in pro-myelocytic leukemia-retinoic acid receptor a (PML-RARα)-positive acute pro-myelocytic leukemia and F-circM9 expression is present in MLL-AF9-positive acute myeloid leukemia. The expression of F-circPR and F-circM9 results in increased cell proliferation and transformation [5].

Our experiments have revealed that there was a fusion circular RNA present in EML4-ALK1-positive lung cancer cells. Using sequencing of PCR products, we have confirmed that F-circEA1 is formed by the reverse connection exons 12–13 of EML4, with exon 20–26 of ALK. Furthermore, research has also found that F-circEA1 promoted tumor cell proliferation, migration, and invasion; these functions were not dependent on the existence of the EML4-ALK1 fusion gene, and was also involved in the regulation of the cell cycle and apoptosis.

Treatment with crizotinib, an inhibitor of anaplastic lymphoma kinase (ALK), promoted drug sensitivity in H3122 cells after interference with F-circEA1, revealing that F-circEA1 is resistant to crizotinib. In vivo, studies have shown that the growth rate of xenogeneic tumors declined and the protein expression level of EML4-ALK1 was significantly decreased in transplanted tumors after this interference by F-circEA1. These results confirmed a role for F-circEA1 in carcinogenesis. To date, many studies have found that ALK fusion genes have obvious carcinogenic potential because the abnormal tyrosine kinase activity enhances cell proliferation and survival, and leads to cytoskeletal rearrangement and morphological changes [18–21]. Our study has confirmed that F-circEA1 positively regulated EML4-ALK1 regardless of mRNA or protein.

Many studies have demonstrated that the downstream signaling pathways affected by EML4-ALK fusion protein are as follows: the RAS-RAF-MEK-ERK pathway which regulates cell proliferation and the PI3K-AKT-mTOR and the JAK3-STAT3 pathways involved in cell survival [22]. In our experiments, F-circEA1 promoted protein expression of the signal factors involved in the downstream pathway of ALK. These findings supported a role for F-circEA1 in the activation of the downstream signaling of ALK, by promoting the expression of EML4-ALK1 and may further influence the biological function of tumor cells. However, the mechanism by which F-circEA1 affects EML4-ALK1 needs to be investigated further. In our experiments, F-circEA1 promoted the protein expression of the main signaling molecules involved in the downstream pathway of ALK. However, it did not affect the mRNA expression of these signaling molecules. It is considered that their expression may be affected at the post-transcriptional or translational levels, or by regulating the expression of EML4-ALK1, which in turn affects the expression of downstream signaling proteins of ALK, which also needs further investigation.

In conclusion, our research has confirmed a direct effect of F-circEA1 on the expression of the parental gene EML4-ALK1, and may activate its downstream signaling pathways. We have also found for the first time, a direct effect of circRNA on the downstream signaling pathways of three of the parental genes and this can further affect the biological function of tumor cells. We have also provided a new role for fusion circRNAs in cancers and that F-circEA1 has potential drug resistance to crizotinib, resulting in a novel treatment for EML4-ALK variant 1 positive NSCLC. However, the mechanism of F-circEA1 affecting EML4-ALK1 needs to be further clarified.

## Supplementary Information

Below is the link to the electronic supplementary material.

Supplementary file1 (DOCX 412 KB)
