# Supplementary material for: F-circEA1 regulates cell proliferation and apoptosis through ALK downstream signaling pathway in non-small cell lung cancer
Source: Hum Cell. 2021 Oct 11;35(1):260–70. doi: 10.1007/s13577-021-00628-7 (PMC8732839; doi:10.1007/s13577-021-00628-7)
Supplement: Supplementary file 1 — Supplementary file1 (DOCX 412 KB) [file 13577_2021_628_MOESM1_ESM.docx]

**Supplementary information, figure s1**


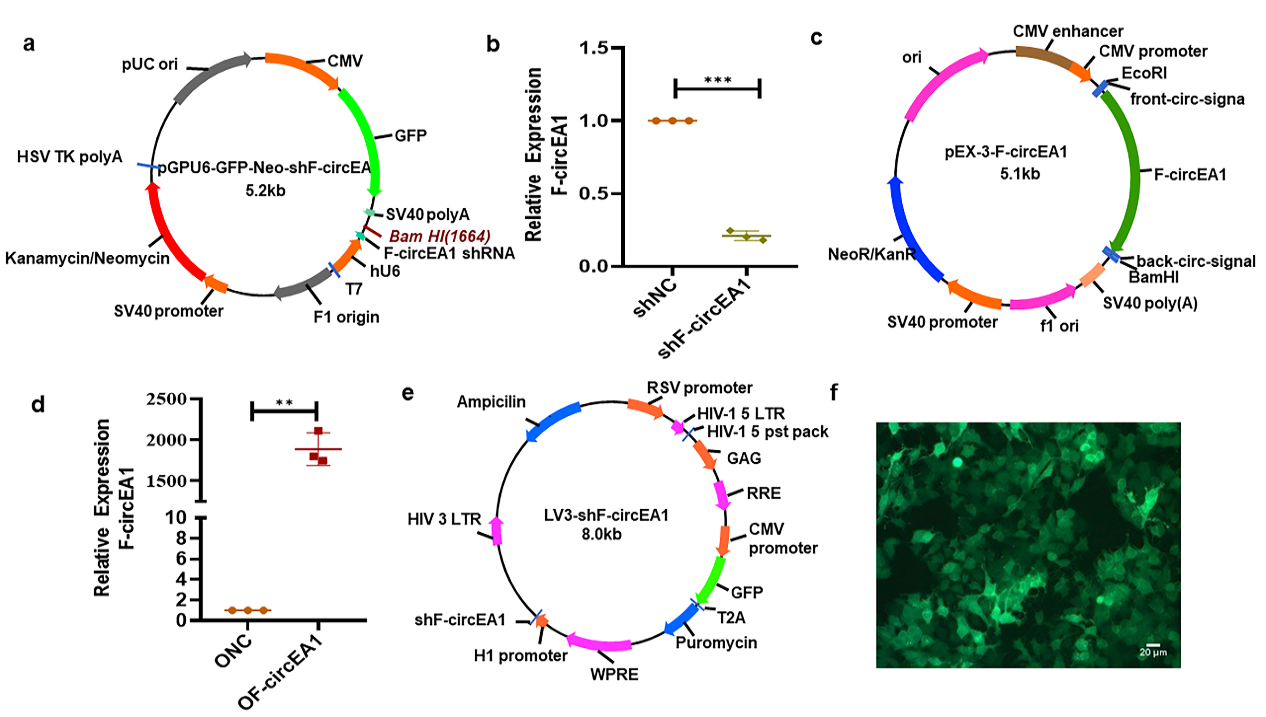


**Fig. s1** a the diagram of the F-circEA1 interference plasmid (pGPU6--GFP-Neo-F-CircEA1). b the expression of F-circEA1 after transfection with the F-circEA1 interference plasmid in H3122. c the diagram of pEX-3-GCMV-F-circEA1-Neo-expression plasmid. d the expression of F-circEA1 after transfection with the F-circEA1 overexpression plasmid in H3122. e Schematic diagram of lentivirus LV3-H1-shF-CircEA1-GFP&Puro and f the transfection diagram in H3122 cells

**Supplementary information, data s1**

Primers and nucleotide sequences used in the study:

**Primers for identification of EML4-ALK1 fusion gene (AB274722.1):**

F1: ATGGACGGTTTCGCCGGC

R1: ACAGGCCCAGGGCAGTTCTT

**Primers for identification of F-circEA1:**

F2: TGTACCGCCGGAAGCACCA

R2: CTTTAGGTCCTTTCCCAGGTGTGGG

**Primers for identification of GAPDH:**

F3: ACCACAGTCCATGCCATCAC

R3: TCCACCACCCTGTTGCTGTA

**qPCR primers for EML4-ALK1:**

F4: GTGCAGTGTTTAGCATTCTTGGGG

R4: TCTTGCCAGCAAAGCAGTAGTTGG

**qPCR primers for F-circEA1:**

F5: CCCCAGAGGCCTTCATGGAA

R5: AGTGAATTGCCGCTCCAGGT

**qPCR primers for GAPDH:**

F6: GAAGGTGAAGGTCGGAGTC

R6: GAAGATGGTGATGGGATTTC

**qPCR primers for PI3K:**

F: ACCACTACCGGAATGAATCTCT

R: GGGATGTGCGGGTATATTCTTC

**qPCR primers for AKT:**

F: GTCATCGAACGCACCTTCCAT

R: AGCTTCAGGTACTCAAACTCGT

**qPCR primers for mTOR:**

F: GCAGATTTGCCAACTATCTTCGG

R: CAGCGGTAAAAGTGTCCCCTG

**qPCR primers for JAK3:**

F: CCTGATCGTGGTCCAGAGAG

R: GCAGGGATCTTGTGAAATGTCAT

**qPCR primers for STAT3:**

F: ACCAGCAGTATAGCCGCTTC

R: GCCACAATCCGGGCAATCT

**qPCR primers for MEK:**

F: CCACGTCATTGCCGTTAAGC

R: GCACGATGTAGGGGCAGTC

**qPCR primers for ERK:**

F: ATGTCATCGGCATCCGAGAC

R: GGATCTGGTAGAGGAAGTAGCA

**shRNA for F-circEA1 knockdown**

AAACAGACACATGACAACAAA

**F-circEA1 full-length sequence**

TGTACCGCCGGAAGCACCAGGAGCTGCAAGCCATGCAGATGGAGCTGCAGAGCCCTGAGTACAAGCTGAGCAAGCTCCGCACCTCGACCATCATGACCGACTACAACCCCAACTACTGCTTTGCTGGCAAGACCTCCTCCATCAGTGACCTGAAGGAGGTGCCGCGGAAAAACATCACCCTCATTCGGGGTCTGGGCCATGGAGCCTTTGGGGAGGTGTATGAAGGCCAGGTGTCCGGAATGCCCAACGACCCAAGCCCCCTGCAAGTGGCTGTGAAGACGCTGCCTGAAGTGTGCTCTGAACAGGACGAACTGGATTTCCTCATGGAAGCCCTGATCATCAGCAAATTCAACCACCAGAACATTGTTCGCTGCATTGGGGTGAGCCTGCAATCCCTGCCCCGGTTCATCCTGCTGGAGCTCATGGCGGGGGGAGACCTCAAGTCCTTCCTCCGAGAGACCCGCCCTCGCCCGAGCCAGCCCTCCTCCCTGGCCATGCTGGACCTTCTGCACGTGGCTCGGGACATTGCCTGTGGCTGTCAGTATTTGGAGGAAAACCACTTCATCCACCGAGACATTGCTGCCAGAAACTGCCTCTTGACCTGTCCAGGCCCTGGAAGAGTGGCCAAGATTGGAGACTTCGGGATGGCCCGAGACATCTACAGGGCGAGCTACTATAGAAAGGGAGGCTGTGCCATGCTGCCAGTTAAGTGGATGCCCCCAGAGGCCTTCATGGAAGGAATATTCACTTCTAAAACAGACACATGACAACAAATGAAGTTGTTTTGGCTGTGGAGTTTCACCCAACAGATGCAAATACCATAATTACATGCGGTAAATCTCATATTTTCTTCTGGACCTGGAGCGGCAATTCACTAACAAGAAAACAGGGAATTTTTGGGAAATATGAAAAGCCAAAATTTGTGCAGTGTTTAGCATTCTTGGGGAATGGAGATGTTCTTACTGGAGACTCAGGTGGAGTCATGCTTATATGGAGCAAAACTACTGTAGAGCCCACACCTGGGAAAGGACCTAAAG
